# EGF induces epithelial-mesenchymal transition and cancer stem-like cell properties in human oral cancer cells via promoting Warburg effect

**DOI:** 10.18632/oncotarget.13771

**Published:** 2016-12-01

**Authors:** Qilin Xu, Qunzhou Zhang, Yasutaka Ishida, Souren Hajjar, Xudong Tang, Haoran Shi, Chi V. Dang, Anh D. Le

**Affiliations:** ^1^ Department of Oral and Maxillofacial Surgery and Pharmacology, University of Pennsylvania School of Dental Medicine, Philadelphia, Pennsylvania, USA; ^2^ Department of Molecular Oral Medicine and Maxillofacial Surgery, Graduate School of Biomedical Sciences, Hiroshima University, Japan; ^3^ Institute of Biochemistry and Molecular Biology, Guangdong Medical University, Zhanjiang, Guangdong, China; ^4^ Abramson Cancer Center, Perelman School of Medicine at The University of Pennsylvania, Philadelphia, Pennsylvania, USA; ^5^ Department of Oral & Maxillofacial Surgery, Penn Medicine Hospital of The University of Pennsylvania, Philadelphia, Pennsylvania, USA

**Keywords:** EGF, EMT, cancer stem cells, Warburg effect, oral cancer

## Abstract

“Warburg effect”, the enhanced glycolysis or aerobic glycolysis, confers cancer cells the ability to survive and proliferate even under stressed conditions. In this study, we explored the role of epidermal growth factor (EGF) in orchestrating Warburg effect, the epithelial-mesenchymal transition (EMT) process, and the acquisition of cancer stem-like cell properties in human oral squamous cell carcinoma (OSCC) cells. Our results showed that EGF induces EMT process in OSCC cells, which correlates with the acquisition of cancer stem-like properties, including the enrichment of CD44^+^/CD24^−^ population of cancer cells and an increased expression of CSC-related genes, aldehyde dehydrogenase-1 (ALDH1) and Bmi-1. We also showed that EGF concomitantly enhanced L-lactate production, while blocking glycolysis by 2-deoxy-D-glucose (2-DG) robustly reversed EGF-induced EMT process and CSC-like properties in OSCC cells. Mechanistically, we demonstrated that EGF promoted EMT process and CSC generation through EGFR/PI3K/HIF-1α axis-orchestrated glycolysis. Using an orthotopic tumor model of human OSCC (UM-SCC1) injected in the tongue of BALB/c nude mice, we showed that treatment with 2-DG *in vivo* significantly inhibited the metastasis of tumor cells to the regional cervical lymph nodes and reduced the expression of ALDH1 and vimentin in both *in situ* tumors and tumor cell-invaded regional lymph nodes. Taken together, these findings have unveiled a new mechanism that EGF drives OSCC metastasis through induction of EMT process and CSC generation, which is driven by an enhanced glycolytic metabolic program in OSCC cells.

## INTRODUCTION

Head and neck squamous cell carcinoma (HNSCC) is one of the 10 most common cancers in the world with more than half a million new cases per year globally, while oral squamous cell carcinoma (OSCC) represents the majority of HNSCC with ∼25,000 new cases diagnosed each year in the U.S. alone [1]. Over half of the patients with head and neck cancer present with locally advanced disease manifested as regional and distant metastases, which contributes to a considerable proportion of the treatment failures and consequently, a ∼50% reduction of survival rates [2–4]. Despite advances in treatment, the 5-year survival still remains disappointing and most of the patients with recurrent or metastatic disease die within a year [4, 5]. An increasing understanding of the complex metastases/recurrence mechanisms of head and neck cancer is imperative to the development of effective and mechanism-based therapeutic modalities for this malignancy.

Epidermal growth factor receptor (EGFR) is a member of the ERBB family of cell-surface tyrosine kinases [6]. It has been reported that more than 90% of head and neck squamous cell carcinomas (HNSCC) overexpress epidermal growth factor receptors (EGFRs), which play an important role in tumor progression and treatment resistance and emerge as important targets for the treatment of HNSCC [4, 7]. Two classes of agents have been explored as specific inhibitors of EGFRs, monoclonal antibodies directed against the extracellular domain of EGFRs and synthetic small molecule tyrosine kinase inhibitors (TKIs) that act directly on the cytoplasmic domain of EGFR TK activity [8]. However, the enthusiasm with EGFR-targeted therapies has been confronted with challenges associated with *de novo* and acquired resistance [6, 9, 10]. Therefore, EGFR-targeted therapies are usually combined with either chemo- or radiation therapies due to the unsatisfactory response rates (∼13%) as a monotherapy [11, 12].

Cancer cells in the primary tumor can lose cell-cell adhesion and break through the basement membrane with increased invasive properties and enter the bloodstream through extravasation, a process driven by epithelial-mesenchymal transition (EMT) process. The circulating tumor cells then exit the bloodstream to form micrometastases, where they undergo mesenchymal-epithelial transition (MET) for clonal outgrowth. Thus, EMT and MET constitute the initiation and completion of the invasion-metastasis cascades. However, the cellular and molecular signals within the tumor microenvironment that orchestrate this complex process are still largely unknown [13]. Cancer stem cells (CSCs) or tumor initiating cells (TICs) represent a small subpopulation of tumor cells that may play a critical role in cancer recurrence, relapse, and metastasis due to their highly tumorigenic, self-renewal, and differentiation capabilities [14]. CSC-like cells have also been identified in head and neck cancer based on the expression of different cellular markers [15–20]. Several lines of evidence have shown that CSCs represent a plastic state of tumor cells undergoing EMT process triggered by various cell-intrinsic or microenvironmental signals [21, 22], however, the exact origin of these unique stem-like cancer cells remains largely unknown. The inherent plastic property of CSCs further supports the notion that even specifically targeting CSCs alone may not be effective to eradicate cancer; thus, multiple combination modalities are necessary to target both CSCs and their unique microenvironment [14]. Accumulating evidence has shown that cancer cells have the ability to rewire their glucose metabolism and energy supply toward glycolysis even in the presence of oxygen, a phenomenon termed Warburg effect or aerobic glycolysis [23, 24]. The aberrant metabolic reprogramming, particularly an increased glycolytic metabolism, can facilitate cancer cells to undergo EMT process and acquire CSC-like properties, thus promoting tumor initiation and progression [25–27]. Therefore, reversing the aberrant metabolic reprogramming of cancer cells is a potential therapeutic approach for cancer therapies [28, 29].

Several lines of evidence have demonstrated that EGF can induce EMT in various types of cancer cells, including breast cancer [30], prostate cancer [31, 32], cervical cancer [33], and head and neck cancer [22, 34]. Meanwhile, EGF stimulation endows head and neck cancer cells with stem-like cell properties [22]. However, the molecular mechanisms underlying EGF-induced CSC phenotypes remain elusive. In the current study, we investigated the potential role of glucose metabolic reprogramming in EGF-induced EMT and cancer stem-like properties in OSCC cells. We showed that EGF enhanced L-lactate production while blocking glycolysis by 2-DG robustly reversed EGF-induced EMT process and CSC-like phenotypes in OSCC cells. Importantly, we demonstrated that *in vivo* treatment with 2-DG significantly inhibited metastasis of tumor cells to regional lymph nodes and robustly reduced the expression of EMT- and CSC-related genes in both the *in situ* tumors and invaded regional lymph nodes. These findings suggest that EGF promotes OSCC metastasis through induction of EMT and CSC generation, which is driven by an enhanced glycolytic metabolic program in OSCC cells.

## RESULTS

### EGF induces EMT process in OSCC cells

**Figure 1:**
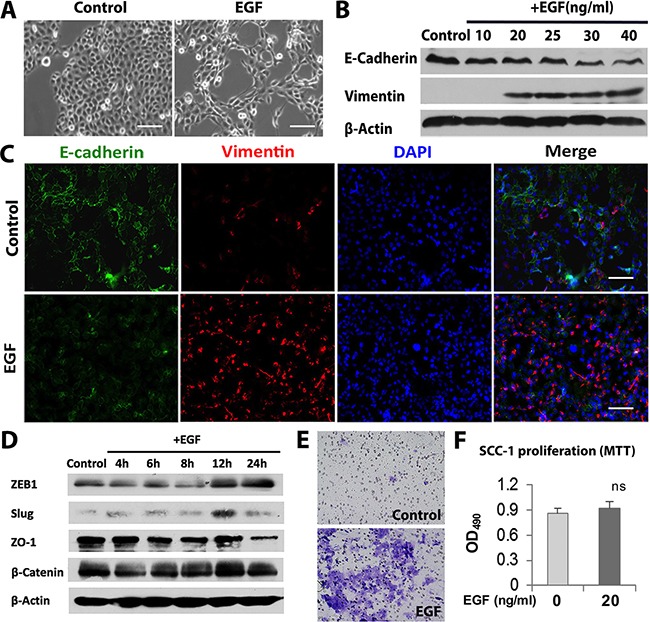
EGF induces EMT process in OSCC cells **A**. Morphologic changes in SCC-1 cells in response to EGF stimulation for 48h. Scale bars, 100μm. **B**. The expression of vimentin and E-cadherin proteins in SCC-1 cells treated with different concentrations of EGF for 48h was determined by Western blot, while the expression of β-actin was used as internal controls. **C**. Immunofluorescence staining of vimentin (Red) and E-cadherin (Green) in SCC-1 cells stimulated with 20ng/mL EGF for 48h was observed under a fluorescence microscope. The nuclei were counter stained with 4′, 6-diamidino-2-phenylindole (DAPI). Scale bars, 100μm. **D**. The expression of ZEB1, Slug, ZO-1 and β-Catenin proteins in SCC-1 cells treated with 20ng/mL EGF for different time periods was determined by Western blot, while the expression of β-actin was used as internal controls. **E**. Stimulation with 20ng/mL EGF for 48h enhanced the invasiveness of SCC-1 cells as evaluated by Matrigel invasion assay. **F**. EGF (20ng/mL) stimulation for 48h showed no obvious effects on cell proliferation of SCC-1 cells as determined by MTT assay. ns, no significant statistical difference.

Initially, we determined the effect of EGF on a panel of established OSCC cell lines and found that two representative cell lines, SCC-1 and SCC-116, underwent typical mesenchymal-like morphological changes characteristic of the EMT phenotype in response to EGF stimulation (Supplementary Figure 1, Figure 1A). EGF-induced EMT process in SCC-1 cells was further confirmed by a dose-dependent decrease in the expression of E-cadherin, a specific cell surface marker for epithelial cells, along with a simultaneous increase in the expression of vimentin, a mesenchymal-related marker (Figure 1B and 1C). The EGF-induced EMT process was also characterized by an increased expression of EMT-regulatory transcription factors, such as Zeb1 and slug, and a decrease in ZO-1, another epithelial cell marker (Figure 1D). Knocking down the expression of Slug and Zeb1 partially abrogated the downregulation of E-cadherin expression and completely abolished the upregulation of vimentin expression induced by EGF in SCC-1 cells (Supplementary Figure 2), suggesting that Slug and Zeb1 contribute to EGF-induced EMT process in SCC-1 cells. Meanwhile, we showed that EGF significantly increased the invasive capacity of SCC-1 cells (Figure 1E), but had little effects on their proliferation rate (Figure 1F), thus ruling out the possibility that the increased number of invasive cells was due to EGF-mediated effect on SCC-1 cell proliferation. These findings suggest that EGF induces EMT process and promotes invasion of SCC-1 cells *in vitro*.

### EGF endows OSCC cells with stem-like cell properties

**Figure 2:**
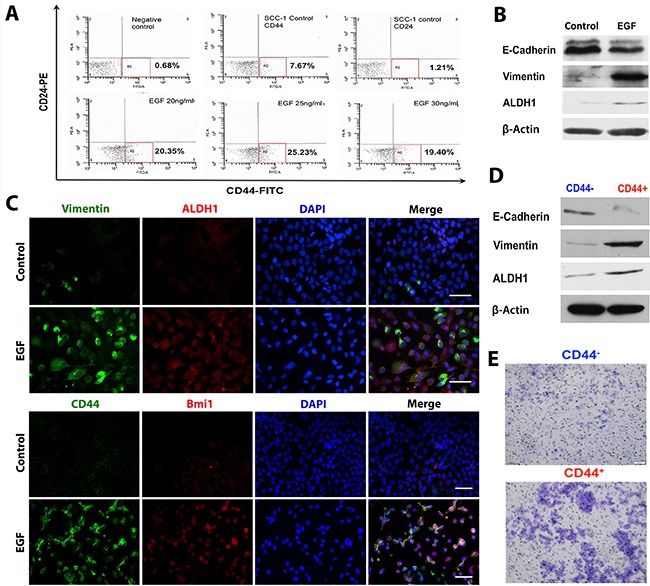
EGF endows OSCC cells with stem-like cell properties **A**. SCC-1 cells were stimulated with different concentrations of EGF for 48h and immunostained with FITC-conjugated anti-human CD44 and PE-conjugated anti-human CD24 antibodies and subjected for flow cytometric analysis. **B**. and **C**. The expression of vimentin, ALDH1, CD44 and Bmi1 in SCC-1 cells stimulated with 20ng/mL EGF for 48h was determined by dual-color immunofluorescence staining (***B***) and Western blot analysis (***C***). The nuclei were counter stained with 4′, 6-diamidino-2-phenylindole (DAPI). Scale bars, 50μm. **D**. The expression of vimentin, E-cadherin, and ALDH1 in purified CD44^+^ and CD44^−^ cells was determined by Western blot, while the expression of β-actin was used as internal controls. **E**. Cell invasiveness of purified CD44^+^ and CD44^−^ cells in the absence of EGF stimulation was determined by Matrigel invasion assay.

We next determined the effect of EGF on CSC-like cell properties in SCC-1 cells. Flow cytometric analysis showed that a small population of SCC-1 cells (∼7-8%) was positive for CD44 (Figure 2A), a commonly used cell surface marker for head and neck cancer stem-like cells [19, 22], while EGF stimulation upregulated CD44 expression and significantly enriched the fraction of CD44^+^/CD24^low/−^ cells (Figure 2A). In addition, EGF not only increased vimentin expression but also significantly enhanced the expression of aldehyde dehydrogenase 1 (ALDH1) and Bmi-1 (Figure 2B and 2C), another two stem cell-related genes popularly expressed in CSC-like cells in HNSCC [19, 22]. We then determine whether EGF-induced CSC-like cells endowed with EMT phenotypes. To this purpose, CD44^+^/CD24^low/−^ CSC-like cells were purified from EGF-treated SCC-1 cells and exhibited a decreased expression of E-cadherin and a concomitant increased expression of vimentin as compared to their counterparts, CD44^−^/CD24^low/−^ cells (Figure 2D). Functionally, we showed that CD44^+^/CD24^low/−^ CSC-like cells intrinsically exhibited increased invasion ability as compared with their CD44^−^/CD24^low/−^ counterparts in the absence of EGF stimulation (Figure 2E). Collectively, these findings suggest that EGF-induced EMT process may play a role in the generation of CSC-like cells with increased invasive capability.

### EGF-induced EMT and CSC-like cell properties depend on aerobic glycolysis

**Figure 3:**
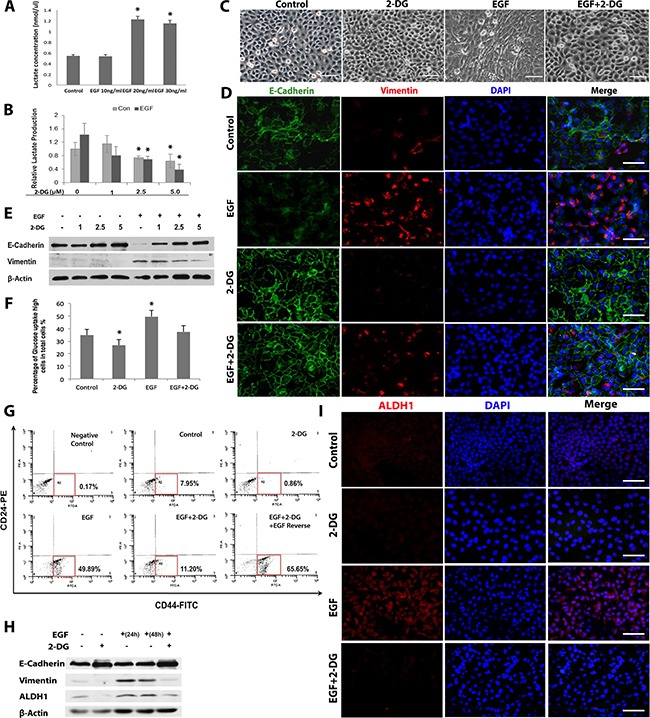
Glycolysis promotes EMT process and generation of CD44+/CD24^low/−^ CSC-like cells in OSCC cells **A**. The lactate production in the supernatants of SCC-1 cells after stimulated with different concentrations of EGF for 24h was determined using a Lactate Assay kit. **B**. SCC-1 cells were stimulated with 20ng/mL EGF in the presence or absence of 5mM 2-DG for 24h and the lactate production in the supernatants was analyzed. *P<0.05. **C**. SCC-1 cells were stimulated with 20ng/mL EGF in the presence or absence of 5mM 2-DG for 48h and cell morphological changes were observed under a microscope. Scale bars, 100μm. **D**. The combinatorial expression of E-cadherin (Green) and vimentin (Red) in SCC-1 cells stimulated with 20ng/mL EGF for 48h in the presence or absence of 5mM 2-DG was determined by dual-color immunofluorescence staining and observed under a fluorescence microscope. The nuclei were counter stained with 4′, 6-diamidino-2-phenylindole (DAPI). Scale bars, 50μm. **E**. EGF-induced downregulation of E-cadherin expression and up-regulation of vimentin expression in SCC-1 cells were reversed by 2-DG treatment as determined by Western blot, while the expression of β-actin was used as internal controls. **F**. The uptake of glucose in SCC-1 cells stimulated with 20ng/mL EGF in the presence or absence of 5mM 2-DG was determined by using a Glucose Uptake Cell-based Assay Kit. *P<0.05. **G**. 2-DG treatment reduced the proportion of CD44^+^/CD24^low^ cells in SCC-1 cells with or without EGF stimulation as determined by flow cytometry. **H**. The downregulation of E-cadherin expression and the upregulation of vimentin and ALDH1 expressions induced by stimulation of SCC-1 cells with 20ng/mL EGF for 24h were reversed by 2-DG treatment for another 24h as determined by Western blot, while the expression of β-actin was used as internal controls. **I**. The increased expression of ALDH1 in SCC-1 cells induced by stimulation with 20ng/mL EGF for 48h was abolished by the presence of 5mM 2-DG as determined by immunofluorescence studies. The nuclei were counter stained with 4′, 6-diamidino-2-phenylindole (DAPI). Scale bars, 50μm.

We then determined the effect of EGF on glycolysis [28, 35] in SCC-1 cells. Our results showed a two-fold increase in the production of lactate in the supernatants of SCC-1 cells after EGF stimulation for 24h (Figure 3A). As expected, pretreatment of SCC-1 cells with various concentrations of 2-deoxy-glucose (2-DG), a glycolysis inhibitor, reduced both the basal and EGF-stimulated lactate production in a dose-dependent manner (Figure 3B). To determine the correlation of EGF-induced EMT and glycolysis, SCC-1 cells were stimulated with EGF in the presence or absence of 2-DG for 48h. Our results showed that the presence of 2-DG significantly prevented mesenchymal-like cell morphological changes in EGF-stimulated SCC-1 cells (Figure 3C). Concurrently, EGF-induced down-regulation of E-cadherin and up-regulation of vimentin expressions were reversed by 2-DG treatment as determined by immunofluorescence studies and Western blot analysis, respectively (Figure 3D and 3E). Meanwhile, EGF-stimulated SCC-1 cells showed a significant increase in glucose uptake as compared to untreated cells, which was also robustly attenuated by 2-DG (Figure 3F). Using another OSCC cell line, SCC-116, we also showed that EGF induced EMT phenotypes characterized by spindle-like cell morphological changes, a downregulation of E-Cadherin expression and a concomitant upregulation of vimentin, Zeb, and Slug expressions, which correlated with an increased expression of stem cell-related genes, ALDH1 and Bmi-1 (Supplementary Figure 3A and 3B). Similarly, EGF significantly increased the lactate production (Supplementary Figure 3D), whereas treatment with 2-DG reversed EGF-induced EMT phenotypes in SCC-116 cells (Supplementary Figure 3C and 3E). These findings suggest that EGF-induced EMT process depends on glycolysis in OSCC cells.

Next, we tested whether glycolysis is also essential to the acquisition of CSC-like cell properties in OSCC cells. Flow cytometric analysis showed that 2-DG treatment markedly reduced the percentage of CD44^+^/CD24^low/−^ subpopulation, from 7.9% to 0.86% in the parental SCC-1 cells and from 49.9% to 11.2% in EGF-pretreated SCC-1 cells, respectively (Figure 3G). Of note, the downregulation of E-cadherin expression and the upregulation of vimentin and ALDH1 expressions induced by stimulation of SCC-1 cells with 20ng/mL EGF for 24h were reversed by 2-DG treatment for another 24h (Figure 3H and 3I). Interestingly, following the withdrawal of 2-DG, re-stimulation of SCC-1 cells with EGF in fresh medium restored the percentage of CD44^+^/CD24^low/−^ subpopulation; while during this reversal EMT process, the heterogeneous cancer cells may undergo non-synchronous morphological changes (mesenchymal and epithelial-like morphologies), thus making them shown as two groups on the dot plots of flow cytometric analysis (Figure 3G; the lower right panel). These results further support the notion that glycolysis may have driven EGF-induced EMT process and CSC-like cell generation in oral cancer cells.

### EGF promotes glycolysis/EMT/CSC-like cell formation through EGFR/PI3K signaling pathways

**Figure 4:**
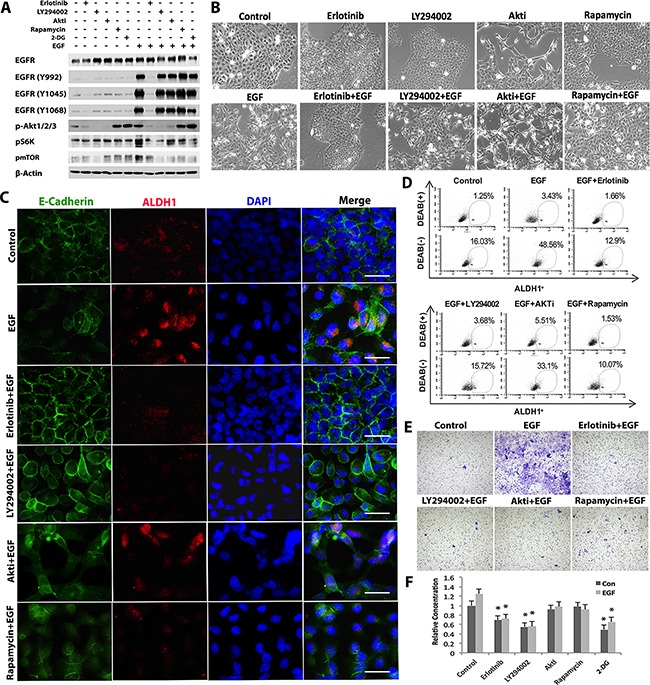
EGF promotes glycolysis/EMT/CSC formation through EGFR/PI3K/mTOR signaling pathway in OSCC cells **A**. SCC-1 cells were pretreated with specific inhibitors for EGFR (Erlotinib), PI3K (LY294002), Akt (AKTi), mTOR (rapamycin) or 2-DG for 1h followed by stimulation with or without EGF for 4h and the expression of EGFR, phosphorylated EGFRs, p-Akt, pS6K, and p-mTOR was determined by Western blot. The expression of β-actin was used as internal controls. **B**. Following similar treatment as describe in (***A***), the morphological changes in SCC-1 cells were observed under a microscope. Scale bars, 100μm. **C**. Following similar treatment as describe in (***A***), the combinatorial expressions of ALDH1 (Red) and E-cadherin (Green) in SCC-1 cells was determined by immunofluorescence studies and observed under a fluorescence microscope; the nuclei were counter stained with 4′, 6-diamidino-2-phenylindole (DAPI). Scale bars, 20μm. **D**. Following similar treatment as describe in (***A***), SCC-1 cells were collected, stained with an ALDEFLUOR™ Kit, and analyzed by flow cytometry. **E**. The effects of various inhibitors of EGFR-mediated signaling pathways on EGF-stimulated invasiveness of SCC-1 cells were evaluated by using Matrigel invasion assay. **F**. Following similar treatment as describe in (***A***), the lactate production in the supernatants of SCC-1 cells was determined using a Lactate Assay kit. *P<0.05.

We then explored whether EGFR-activated signaling pathways are involved in the regulation of EGF-induced glycolysis/EMT/CSC-like cell generation. As expected, EGF stimulation resulted in EGFR phosphorylation at several specific tyrosine residues (including Tyr992, 1045, 1068) and correspondingly, the activation of its downstream signaling pathways, including pAkt, pS6Kp70, and mTOR, which was completely blocked by erlotinib, a specific EGFR tyrosine kinase inhibitor (TKI) (Figure 4A). Blocking PI3K activity by LY294002 completely abolished EGF-induced phosphorylation/activation of Akt, pS6K70, and mTOR (Figure 4A); treatment with AKT inhibitor (AKTi) blocked EGF-induced phosphorylation of both Akt and mTOR, but had little effects on pS6Kp70, while rapamycin, a specific inhibitor of mTOR, completely blocked EGF-induced mTOR activation but without any obvious effects on Akt and pS6Kp70 activation (Figure 4A). These findings suggest that EGF exerts its biological effects through the activation of EGFR/PI3K/Akt/mTOR signaling cascades in SCC-1 cells.

Next, we determined the role of EGFR signaling pathways in the regulation of EGF-induced EMT process and CSC-like phenotypes in SCC-1 cells. We found that blocking EGFR, PI3K or mTOR activities with erlotinib, LY294002 or rapamycin significantly abrogated EGF-induced mesenchymal-like morphological changes and the downregulation of E-cadherin expression in SCC-1 cells, while the AKT inhibitor showed no obvious effects (Figure 4B and 4C). Additionally, blocking EGFR, PI3K or mTOR activities, but not AKT activation, also markedly attenuated EGF-induced up-regulation of ALDH1 expression in SCC-1 cells as determined by immunofluorescence staining and flow cytometric analysis (Figure 4C and 4D). Functionally, we showed that blockade of EGFR/PI3K/AKT/mTOR signaling pathways significantly abrogated EGF-induced invasive abilities of SCC-1 cells (Figure 4E). Similar to 2-DG treatment, blocking either EGFR or PI3K activity significantly attenuated EGF-stimulated lactate production in SCC-1 cells (Figure 4F). Taken together, these findings suggest that EGF induces glycolysis/EMT/CSC-like cell properties in SCC-1 cells via EGFR/PI3K/mTOR-dependent but AKT-independent mechanisms. Further studies are warranted to delineate the detailed mechanisms that integrate EGF-induced glycolysis, EMT process and CSC-like cell generation.

### EGF induces EMT process via EGFR/PI3K signaling-mediated PDK1 expression in SCC-1 cells

**Figure 5:**
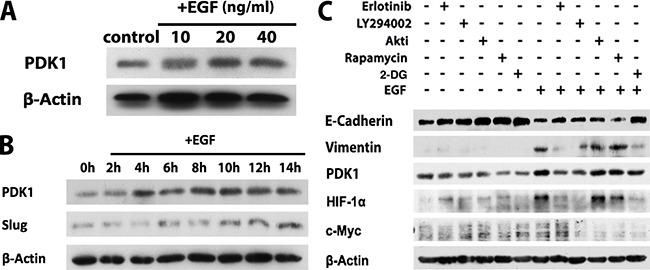
EGFR/PI3K/HIF-1α signaling pathways facilitate glycolysis through promoting PDK1 expression in OSCC cells **A**. The expression of PDK1 in SCC-1 cells following treatment with different concentrations of EGF for 24h were determined by Western blot. **B**. The expression of PDK1 and Slug in SCC-1 cells following treatment with 20ng/mL EGF for different time periods was determined by Western blot. **C**. SCC-1 cells were pretreated with specific inhibitors for EGFR (Erlotinib), PI3K (LY294002), Akt (AKTi), mTOR (rapamycin), or 2-DG for 1h followed by stimulation with or without EGF for 24h, and the expression of E-cadherin, Vimentin, PDK1, HIF-1α, and c-Myc was determined by Western blot. The expression of β-actin was used as internal controls.

A recent study has demonstrated the cross-talk between EGFR activation and pyruvate dehydrogenase kinase 1 (PDK1), one of the key metabolic regulators of glycolysis [28]. We then determined the role of EGFR signaling pathways in the regulation of PDK1 expression in OSCC cells. Our results showed that EGF potently induced PDK1 expression in both SCC-1 (Figure 5A and 5B) and SCC-116 (Supplementary Figure 3B). The increase in PDK1 expression in SCC-1 cells started as early as 4 h post EGF stimulation, which happened earlier than EGF-induced upregulation of Slug expression, a key EMT-regulatory transcription factor (Figure 5B). Interestingly, blocking EGFR and PI3K activities, but not the AKT and mTORC1 activities, almost completely abrogated EGF-induced PDK1 upregulation, which was coupled with the reversal of EGF-induced EMT phenotypes in SCC-1 cells, specifically, the downregulated E-cadherin expression and the upregulated expression of vimentin (Figure 5C). Of note, such effects conferred by blockade of EGFR and PI3K activities on EGF-induced PDK1 expression and reversal of EMT phenotypes, were comparable to those mediated by 2-DG treatment (Figure 5C). Meanwhile, our results showed that EGF significantly upregulated HIF-1α protein expression, a transcription factor that cooperates with c-Myc to drive the PDK1 expression and facilitate glycolysis [36–38], which is correlated with EGF-induced upregulation of PDK1 in SCC-1 cells (Figure 5C). However, EGF has no direct effect on c-Myc expression (Figure 5C). Taken together, these findings suggest that EGFR/PI3K/HIF-1α signaling-orchestrated glycolysis may play an important role in EGF-induced EMT process in OSCC cells.

### Blocking glycolysis suppressed EGF-facilitated metastasis of OSCC cells to regional cervical lymph nodes

**Figure 6:**
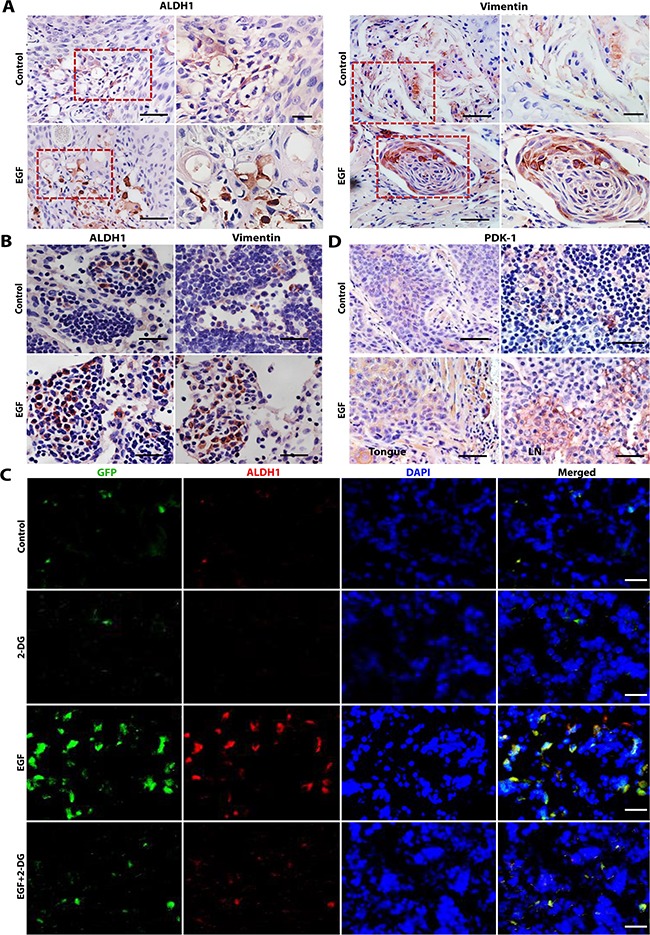
2-DG treatment suppresses EGF-facilitated regional cervical lymph node metastasis in an orthotopic tongue model of OSCCs in nude mice **A**. EGF increased the expression of ALDH1 and vimentin in primary orthotopic SCC-1 tumors in the tongue as determined by immunohistochemical (IHC) studies. The left panels, 10× magnification, Scale bars, 100μm; the right panels, 20× magnification; Scale bars, 50μm. **B**. The expression of vimentin and ALDH1 in metastatic SCC-1 cells at cervical lymph nodes was determined by IHC. Scale bars, 50μm. **C**. The expression of ALDH1 (Red) in metastatic GFP-tagged SCC-1 cells (Green) at cervical lymph nodes was determined by immunofluorescence staining and observed under a fluorescence microscope; the nuclei were counter stained with DAPI. Scale bars, 50μm. **D**. The expression of PDK1 in primary orthotopic tumors in the tongue (the left panels) and the cervical lymph nodes (LN) (the right panels) was determined by IHC staining. Scale bars, 50μm.

We next determined the critical role of glycolysis in EGF-facilitated regional cervical lymph node (LN) metastasis in an orthotopic tumor model of human OSCC in the tongue of nude mice. To this purpose, SCC-1 cells were stably transduced with a GFP-expressing lentiviral vector (Supplementary Figure 4A). These GFP-tagged SCC-1 cells underwent EMT process in response to EGF stimulation, which could also be reversed by 2-DG treatment *in vitro* (Supplementary Figure 4A). Following submucosal injection into the tongue of nude mice, GFP-tagged SCC-1 cells steadily formed tumors (Figure 6A; Supplementary Figure 4B), and about 20% of mice developed regional cervical LN metastasis as evidenced by the local presence of GFP-tagged SCC-1 cells (Supplementary Figure 4C, Table 1). Upon transplantation in a mixture of hydrogel with EGF (20ng/mL), SCC-1 formed tumors *in situ* with similar sizes to those without EGF (Supplementary Figure 4B), suggesting that EGF did not promote the growth of the *in situ* tumors. However, the presence of EGF enhanced the expression of ALDH1, vimentin, and PDK1in the *in situ* tumors in the tongue (Figure 6A and 6D) and increased the incidence of cervical LN metastasis (70% *versus* 20% of the control group; p=0.038) (Fig. S4C, Table 1). Additionally, our results showed that the metastatic tumor cells in the cervical LNs positively expressed vimentin, ALDH1 (Figure 6B and 6C), and PDK1 (Figure 6D), suggesting they were endowed with EMT and CSC-like phenotypes. Importantly, our data indicated that 2-DG treatment had no obvious inhibitory effects on the *in situ* tumor growth (Supplementary Figure 4B), but dramatically reduced the degree and incidence of EGF-facilitated cervical LN metastases of SCC-1 cells (30% *versus* 70%) even though there was no significant statistic difference (Figure 6C, Table 1 ; p=0.101). Collectively, these *in vivo* findings support that glycolysis plays a pivotal role in EGF-facilitated metastasis of OSCC cells.

**Table 1:** Incidence of cervical lymph node (LN) metastasis

	Groups	No. of Mice	No. of Mice with LN Metastasis	Percentage (%)
**I**	SCC1	10	2	20.0
**II**	SCC1+2-DG	10	0	0
**III**	SCC1 + EGF	10	7	70.0*
**IV**	SCC1+EGF+2-DG	10	3	30.0^#^
			*P=0.038 (III *v.s*. I)	^#^*P*=0.101 (IV *v.s*. III)

## DISCUSSION

The epithelial-mesenchymal transition (EMT) process constitutes a crucial step in the invasion and metastatic spread of a variety of epithelial cancers [14]. This phenotypic transition process is dynamic, reversible and bidirectional (EMT *vs*. MET), thus contributing to the plastic states of cancer cells. The plasticity of cancer cells is controlled by both intrinsic biochemical processes and extrinsic bi-directional cues derived from cancerous and non-cancerous cells in tumor microenvironment [14]. Increasing evidence supports the notion that the plastic property of neoplastic non-stem cells allows them to be reprogrammed into a more primitive, stem-like phenotype [14, 39]. From this point of view, CSCs may not be a distinct cellular entity but rather represent a state of tumor cells that transiently acquire stem cell-like properties as a consequence of EMT process, thus contributing to the uncontrollable metastasis, relapse, and therapeutic resistance. Therefore, a better understanding of the complex network that orchestrates the EMT process may hold a great promise for the development of novel therapeutic approaches to eradicate various types of cancers that are currently resistant to conventional therapies.

EGF, one of the most abundant growth factors found in the tumor microenvironment, can be produced by cancer cells *per se* and non-cancerous cells such as mesenchymal stromal cells [40], endothelial cells [22], and macrophages [41, 42]. The abundance of EGF together with the overexpression of EGFR observed in diverse types of cancers, including head and neck cancer [7, 22, 34], lead to aberrant activation of the downstream signaling pathways that facilitate EMT process [40, 41], which subsequently renders cancer cells with plastic properties and increased invasive and metastatic capabilities [22, 34, 41]. Herein, we have demonstrated that activation of EGF/EGFR signaling pathways facilitates the EMT process and enrichment of ALDH^+^/CD44^high^ CSC-like cells with increased invasiveness/metastasis potentials both *in vitro* and *in vivo*. These findings, together with previous studies by others [22, 34], support the notion that the EMT process and acquisition of CSC-like phenotypes driven by the activated EGF/EGFR signaling cascades may play a critical role in the metastasis and recurrence of OSCC.

Even though EGFR activation leads to aberrant EMT process and acquisition of CSC-like properties under various settings [22, 40, 41], the beneath mechanisms remain largely unknown. Most recently, several studies have demonstrated the relation between EGFR activation and dysregulated Warburg effect or aerobic glycolysis in tumor cells [28, 34, 43, 44]. On one hand, EGFR activation promotes tumorigenesis probably by enhancing glycolysis [35, 45], while blocking EGFR activity leads to the reversal of the Warburg effect [28, 44]. On the other hand, blocking glycolysis sensitizes non-small cell lung cancer cells with a T790M mutation to the treatment with irreversible EGFR inhibitors [43]. Consistently, in the present study we have shown that EGF stimulation significantly enhanced glycolysis in OSCCs possibly by upregulating PDK1 expression and blocking glycolysis almost completely abolished EGF-induced EMT process and acquisition of CSC-like phenotypes in OSCCs both *in vitro* and *in vivo*. These compelling findings support the notion that the dysregulated “Warburg effect” may play a critical role in the EMT process and acquisition of CSC-like phenotype driven by aberrant activation of EGF/EGFR signaling cascades.

Previous studies have shown that over 90% of HNSCC overexpress EGFRs and the majority of them are resistant to conventional chemo- and radiation-therapies [4, 7]. Cetuximab is the only EGFR inhibitor approved for the treatment of HNSCC, but the response rate is low [8]. Therefore, it is usually used in combination with chemo- and radiotherapy for HNSCC [4]. To date, it remains a challenge to unveil the molecular and cellular mechanisms by which HNSCC cells develop the *de novo* and acquired resistance to EGFR-targeted therapies [4, 6]. A better understanding of the metabolic plasticity or dysregulated metabolic reprogramming of cancer cells may help us to elucidate how they contribute to resistance to conventional chemo- and radiation therapies and EGFR-targeted therapies. Recent studies have shown that targeting the unique metabolic properties can specifically eliminate cancer stem cells or tumor initiating cells [46, 47]. Currently, we have provided the first line of evidence that blocking glycolysis using 2-DG remarkably suppressed EGF-facilitated regional cervical LN metastases of OSCCs. All together, these findings suggest that targeting cellular metabolism may become a new paradigm to improve the responses of cancer cells to both conventional and EGFR-targeted therapeutics for HNSCC.

## MATERIALS AND METHODS

### Animals

Six- to eight-week-old female Athymic NCr-nu/nu mice were purchased from NCI (Frederick, MD) and maintained under standard conditions. All animal procedures were handled according to the guidelines of the Institutional Animal Care and Use Committee (IACUC) of the University of Pennsylvania. We adopted a randomized, prospective and controlled animal model design according to all the recommendations of the ARRIVE (Animal Research: Reporting *In Vivo* Experiments) guidelines. Mice were group-housed in polycarbonate cages (5 animals per cage) in the animal facilities with controlled temperature (23°C±2°C), 40–65% of humidity and a 12-hour light/dark cycle. Mice were acclimatized for at least 1 week prior to the study, fed with a standard laboratory diet and allowed *ad libitum* access to drinking water.

### Cell lines and treatment

University of Michigan squamous cell carcinoma (UM-SCC-1), a floor-of-the mouth squamous cell carcinoma derived from tumor recurrence, was kindly provided by Professor Cun-yu Wang (University of California, Los Angeles) and cultured in RPMI-1640 supplemented with 10% fetal bovine serum (FBS), 100IU/ml penicillin, 100μg/ml streptomycin [48]. UPCI: SCC-116, an alveolar ridge SCC, was kindly provided by Professor Susanne M. Gollin (Head and Neck Spore Grant at University of Pittsburgh Cancer Institute) [49] and cultured in MEM medium supplemented with MEM-non-essential amino acid (NEAA) (Invitrogen), gentamycin, and 10% FBS. CAL27 (ATCC® CRL-2095™), a human tongue squamous cell carcinoma cell line, and FaDu (ATCC ® HTB-43™), a human squamous cell carcinoma cell line derived from the hypopharynx, were both obtained from ATCC [50] and cultured in DMEM supplemented with 10% fetal bovine serum (FBS), 100IU/ml penicillin, 100μg/ml streptomycin.

Cells were stimulated with different concentrations of EGF (0∼40ng/ml) (PeproTech) for 48h in the presence or absence of 5mM 2-deoxy-glucose (2-DG) (Sigma), a recognized glycolysis inhibitor. To study downstream EGFR signaling pathway, cells were pretreated with 5μM Erlotinib (Cell Signaling Technology), an EGFR tyrosine kinase inhibitor, 50μM LY294002 (EMD Millipore), a PI3K inhibitor, or 20μM AKT inhibitor (EMD Millipore), or 2.5μM Rapamycin (EMD Millipore), an mTOR inhibitor, in serum-free RPMI for 1h followed by stimulation with EGF for 4h.

### Establishment of stable GFP-expressing cell lines

A pLL3.7 lentiviral GFP vector was a kind gift from Dr. Andy Peng Xiang (Sun Yat-sen University, China) [51]. pMD2.G (VSV-G) envelope plasmid (#12259) and psPAX2 packaging plasmid (#12260) were from Addgene. Lentiviral products were produced by cotransfection of 293FT packaging cells using Lipofectamine 2000. Briefly, 293FT cells were pre-plated in 10-cm tissue culture plates (6.5 × 10^6^ cells/plate) in DMEM with 10% FBS so that they were about 90∼95% confluent on the day of transfection. 293FT cells were cotransfected with 5 μg of pMD2.G (VSV-G) envelope plasmid, 10μg of psPAX2 packaging plasmid and 15μg of lentiviral construct plasmids. 12 h later, the culture medium was replaced with 10 ml fresh complete culture medium. The lentivirus-containing supernatants from the transfected cells were collected 48-72 h after transfection, filtered with 0.45μm Millex-HV filters, aliquoted and stored at –80°C. SCC-1 cells were plated in 6-well plate (1 × 10^5^ cells/well) and transduced overnight with 1ml lentivirus-containing supernatant at a multiplicity of infection (MOI) of 10 in the presence of 8μg/ml polybrene (Sigma) and then remove the culture medium and replace with 2ml of complete medium without polybrene. 48h after transduction, SCC-1 cells were subjected for selection with 2μg/ml puromycin and continuously subcultured. The transduction efficiency was determined by observation under a fluorescence microscope (Olympus XF-73). Meanwhile, the GFP^+^ cells were further sorted using a cell sorting flow cytometer (FACS Aria D; Flow Cytomerty & Cell Sorting Facility, University of Pennsylvania School of Medicine).

### Flow cytometry and cell sorting

Cells were harvested and washed twice with PBS containing 2% heat-inactivated FBS, and resuspended in cell staining buffer (BioLegend, San Diego, CA) at a concentration of 10^7^/ml. Cell suspensions were incubated with PE-CD44 and FITC-CD24 antibodies (BD Biosciences, San Diego, CA), or an isotype-matched mouse IgG control at 4°C for 30min. Then, the samples were washed twice with PBS/2% FBS and submitted to flow cytometric analysis (BD LSRII). For cell sorting, cells were collected and immunostained with PE-CD44 and FITC-CD24 antibodies, or stained with Aldefluor kit (Stem Cell Technologies) using diethylaminobenzaldehyde (DEAB, a specific inhibitor of ALDH activity) as negative control followed by immunostained with PE-CD44 antibody. The stained cells were then submitted for cell sorting using a cell sorting flow cytometer (FACS Aria D).

### Glucose uptake and lactate secretion

Cells were seeded in 6-well flat-bottomed plates at a density of 5 × 10^5^ cells per well and treated with EGF or 2-DG at the indicated concentration in culture medium for 48 h. The glucose uptake was determined by Glucose Uptake Cell-based Assay Kit (Cayman Chemical) following manufactures’ instructions. All cells were analyzed with a flow cytometer. In parallel experiments untreated and treated cells were analyzed for lactate levels in the conditioned media. Briefly, after different treatments, conditioned media were removed, centrifuged at 13000 × g at 4°C for 10 min and then assayed for lactate concentration using the L-lactate assay kit (Eton Biosciences) following manufactures’ instructions. Absolute lactate levels were calculated from the corresponding standard curve and normalized by cell numbers.

### Hematoxylin and eosin (H&E) staining and immunohistochemistry (IHC)

Dissected tumor tissues and metastatic lymph nodes from tumor-bearing nude mice were fixed with 4% PFA and embedded in paraffin. 5-μm thick sections were deparaffinized with xylene and rehydrated in graded ethanol. For the purpose of antigen retrieval, the sections were incubated in sodium citrate buffer (10mM, pH 6.0) at 95°C for 20 min. After blocking with 2.5% goat serum in PBS, the sections were incubated overnight at 4°C with primary antibodies (1:200) for vimentin (rabbit IgG; Cell Signaling Technology, Danvers, MA, USA), ALDH1 (rabbit IgG; Cell Signaling Technology), or PDK-1(rabbit IgG; Enzo Life Sciences, Inc., Farmingdale, NY). For the subsequent steps, sections were stained with VECTASTAIN Universal ABC Elite Kit (PK-7200; Vector Labs, Burlingame, CA) and colors were developed using VECTOR NovaRED Substrate Kit (SK-4000; Vector Labs) according to the manufacture's procedures and briefly counterstained with hematoxylin and observed under a light microscope with a digital camera.

### Immunofluorescence studies

For immunofluorescence staining, 10-μm-thick frozen sections or cultured cells on 8-well chamber slides were fixed in 4% PFA for 10 min at room temperature followed by permeabilization with 0.1% Triton-X100 and blocking with 2.5% goat serum in PBS. Then, samples were incubated at 4°C overnight with primary antibodies for E-cadherin (mouse IgG, Santa Cruz Biotechnology, Santa Cruz, CA), CD44 (mouse IgG; BD Biosciences, San Diego, CA), vimentin (rabbit IgG; Cell Signaling Technology), Bmi-1(rabbit IgG; Santa Cruz Biotechnology), or ALDH1(rabbit IgG; Cell Signaling Technology), followed by incubation with FITC- or rhodamine-conjugated secondary antibodies (1:400; BioLegend, San Diego, CA) at room temperature for 1h. An isotype control IgG was used as nonspecific controls. The nuclei were counterstained with 4′, 6-diamidino-2-phenylindole (DAPI) contained in the mounting medium (Vector Labs) and images were observed under a fluorescence microscope (Olympus XF-73).

### 3D-cell invasion assay

The invasion ability of parental SCC-1 in the presence or absence of 20ng/mL EGF, the sorted CD44^+^/CD24^−^ and CD44^−^/CD24^+^ cells was determined using the QCM^TM^ Collagen-based Cell Invasion Assay Kit according to the manufacturer's protocol (Millipore). Cells were seeded into upper inserts at 1 × 10^5^ per insert in serum-free DMEM. Outer wells were filled with DMEM containing 10% FBS or 20ng/mL EGF as chemoattractants. Cells were incubated at 37°C with 5% CO_2_ for 48h. The non-invading cells were removed by swabbing top layer of collagen with Q-tips, while cells migrated through the gel insert to the lower surface of the membrane were stained and photographed under a microscope at 40 × objectives [20]. To determine the effect of EGF on cell proliferation, SCC-1 cells were seeded into 96-wells (1×10^4^/well in triplicate) and cultured in the presence or absence of 20ng/mL EGF for 48h and the proliferation was determined using [3-(4,5-dimethylthiazol-2-yl)-2,5-diphenyltetrazolium bromide] (MTT) assay.

### siRNA transient transfection

SCC-1 cells (2×10^5^/well) were seeded into 6-well plates and transfected with a pool of 3 target-specific 19-25nt siRNA for SLUG1 (sc-38393; Santa Cruz Biotech) or ZEB1 (sc-156138; Santa Cruz Biotech); a non-specific siRNA (sc-36869, Santa Cruz) was used as control. Following transfection for 5h, the transfection medium was replaced with fresh complete medium and cells were cultured for overnight followed by stimulation with EGF (20ng/ml) for 48h.

### Western blot analysis

Cell lysates (50 μg of total protein) were separated on polyacrylamide-SDS gel and electroblotted onto nitrocellulose membrane (BioRad, Hercules, CA). After blocking with TBS/5% nonfat dry milk, the membrane was incubated with antibodies against E-cadherin (mouse IgG, Santa Cruz Biotechnology), vimentin (mouse IgG, Santa Cruz Biotechnology), ZEB1, ZO-1, Slug, ALDH1, p-Akt (Ser473), EGFR, or p-EGFRs (rabbit IgG; Cell Signaling Technology) followed by incubation with a horseradish peroxidase (HRP)-conjugated secondary antibody, and the signals were visualized by enhanced chemiluminescence detection (ECL) (PIERCE, Rockford, IL). The blots were also re-probed with a specific antibody against β-actin (Santa Cruz Biotechnology).

### An orthotopic OSCC model in the tongue of nude mouse

Subconfluent GFP-tagged SCC-1 cells were harvested, washed and resuspended in PBS. 1 × 10^5^ cells suspended in 30μL of PBS were injected into the lateral tongue as described previously [50, 52]. To observe the effect of EGF on tumor growth and lymph node metastasis, 30μL of Hydrogel containing 1 × 10^5^ GFP-tagged SCC-1 cells with/without EGF (20ng/ml) was injected submucosally into anterior-sublingual portion of tongue. Two days after tongue injection of tumor cells, mice were intraperitoneally (i.p.) administered with 2-DG (500mg/kg) every other day. Mice with orthotopic tumor transplantation treated with the vehicle were used as control (n=10). Body weight was assessed twice a week. After 4 weeks, the mice were sacrificed with CO_2_, and the tongue tumors and cervical lymph nodes were removed, fixed in 4% PFA and processed for further analysis. Tumor volume was calculated as *V* = *AB*^2^(π/6), where *A* is the longest dimension of the tumor and *B* is the dimension of the tumor perpendicular to *A*. The presence of cervical lymph node and distant lung metastasis was evaluated by the presence of GFP signals and the Hematoxylin and eosin (H&E) staining [53–55].

### Statistical analysis

All data are presented as mean ± standard deviation (SD). The Student's paired or independent *t-*tests were used to analyze the differences *in vitro* experimental data [54, 55], while incidence rate of lymph node metastasis was analyzed using Fisher's exact test [56]. A values of *P*<0.05 was considered to be statistically significant. Statistical analyses were performed using SPSS 18.1statistical software program (SPSS Inc., Chicago, IL, USA).

## SUPPLEMENTARY FIGURES


