# Supplementary material for: EGF induces epithelial-mesenchymal transition and cancer stem-like cell properties in human oral cancer cells via promoting Warburg effect
Source: Oncotarget. 2016 Dec 1;8(6):9557–71. doi: 10.18632/oncotarget.13771 (PMC5354753; doi:10.18632/oncotarget.13771)
Supplement: Supplementary file 1 [file oncotarget-08-9557-s001.pdf]

EGF induces epithelial-mesenchymal transition and cancer stem-like cell properties in human oral cancer cells via promoting Warburg effect

SUPPLEMENTARY FIGURES

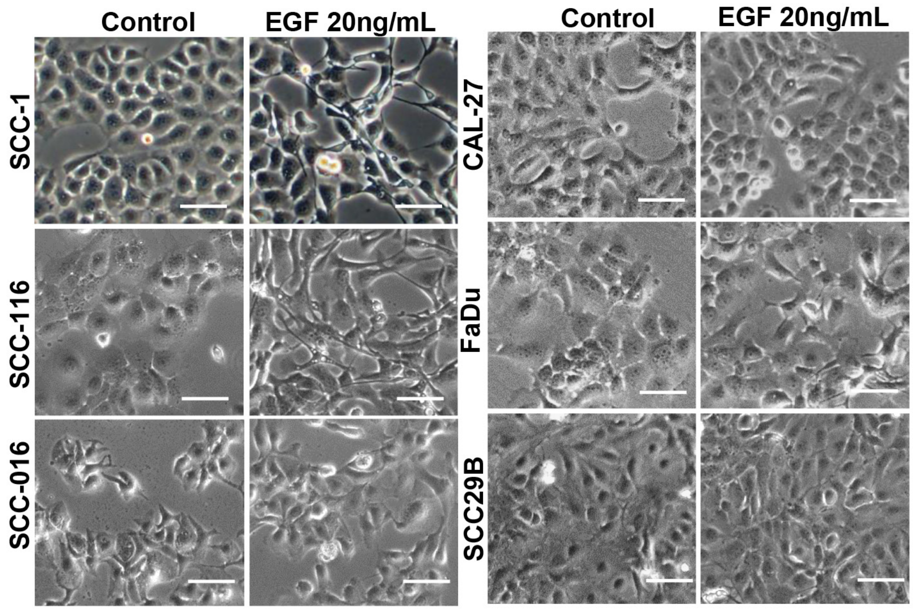

Supplementary Figure 1: Morphological changes in human OSCC cell lines following stimulation with EGF. Human OSCC cell lines, SCC-1, SCC-116, SCC-016, CAL-27, FaDu, and SCC29B, were stimulated with 20ng/mL EGF for 48h, and their morphological changes were observed under a microscope. Following stimulation with 20ng/mL EGF, SCC-1 and SCC-116 cells underwent mesenchymal-like morphological changes. Scale bars, 50µm.

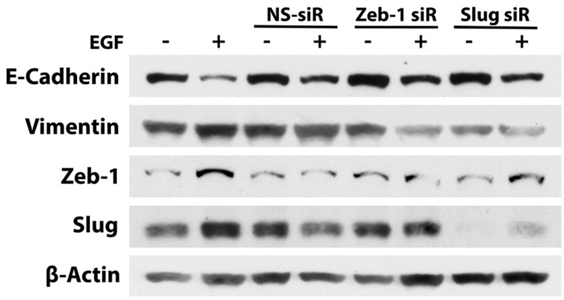

Supplementary Figure 2: The functional role of SLUG and ZEB1 in EGF-induced EMT process in SCC-1 cells. SCC-1 cells were transiently transfected with specific siRNAs targeting Zeb-1 (Zeb-1 siR) or Slug (Slug siR), or a non-specific siRNA (NS-siR) followed by stimulation with or without 20ng/mL EGF for 48h. The expression of E-cadherin, Vimentin, Zeb-1, and Slug was determined by Western blot, while the expression of β-actin was used as internal controls.

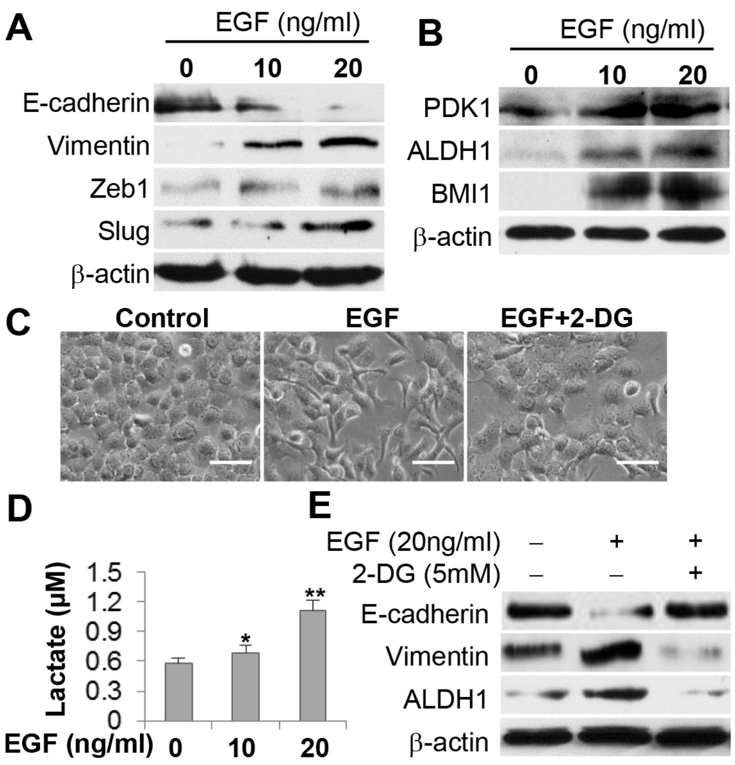

Supplementary Figure 3: EGF facilitates glycolysis and EMT process in SCC-116 cells A. and **B.** SCC-116 cells were stimulated with different concentrations of EGF for 48h and the expression of E-cadherin, vimentin, Zeb-1, Slug, PDK1, ALDH1, and BMI-1 was determined by Western blot, while the expression of β-actin was used as internal controls. **C.** SCC-116 cells were stimulated with 20ng/mL EGF in the presence or absence of 5mM 2-DG for 48h, and the morphological changes were observed under a microscope. Scale bars, 50μm. **D.** SCC-116 cells were stimulated with different concentrations of EGF for 24h and the lactate production in the supernatants was determined using a Lactate Assay kit. $^{*}P<0.05$; $^{**}P<0.01$. **E.**, SCC-116 cells were stimulated with 20ng/mL EGF in the presence or absence of 5mM 2-DG for 48h, and the expression of E-cadherin, vimentin, and ALDH1 was determined by Western blot, while the expression of β-actin was used as internal controls.

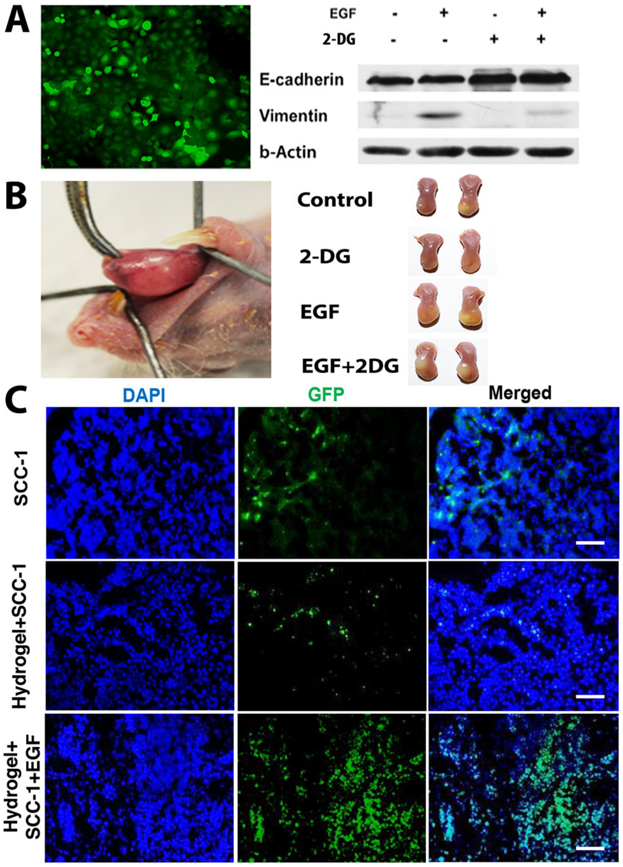

Supplementary Figure S4: EGF facilitates the metastasis of orthotopic SCC-1 tumor cells in the tongue to regional cervical lymph nodes. A. Establishment of SCC-1 cells stably expressing GFP proteins (GFP-tagged SCC-1, the left panel). 2-DG treatment abrogated EGF-induced upregulation of vimentin expression in GFP-tagged SCC-1 cells (the right panel). **B.** GFP-tagged SCC-1 cells steadily formed tumors after orthotopic transplantation into the tongue of nude mice for 4 weeks, while the presence of EGF (20ng/mL) showed no obvious effects on the growth of the *in situ* tumors. Meanwhile, treatment with 2-DG had no obvious inhibitory effects on the growth of the *in situ* tumors. **C.** EGF facilitates metastasis of GFP-tagged SCC-1 cells (Green) to the regional cervical lymph nodes as observed under a fluorescence microscope; the nuclei were counter stained with DAPI. Scale bars, 100µm.
